# Unveiling the contourite depositional system in the Vema Fracture Zone (Central Atlantic)

**DOI:** 10.1038/s41598-023-40401-4

**Published:** 2023-08-24

**Authors:** Dmitrii G. Borisov, Dmitry I. Frey, Elena V. Ivanova, Nikolay N. Dmitrevskiy, Oleg V. Levchenko, Vladimir V. Fomin, Marco Ligi

**Affiliations:** 1 Shirshov Institute of Oceanology, Russian Academy of Sciences, Moscow, Russia; 2 Moscow Institute of Physics and Technology, Dolgoprudny, Russia; 3 Zubov State Oceanographic Institute, Moscow, Russia; 4 Istituto di Scienze Marine, Consiglio Nazionale delle Ricerche, Bologna, Italy

**Keywords:** Geomorphology, Geophysics, Sedimentology

## Abstract

A combination of a high sediment input and intense bottom currents often leads to the formation of contourites (sediments deposited or significantly reworked by bottom currents). Both of these components are present in the Vema Fracture Zone valley which is the most important passageway for the distribution of the Antarctic Bottom Water from the West to the North-East of the Atlantic. However, no contourite drifts, moats or contourite channels have been found in this region in more than half a century of research. The prevailing sedimentation paradigm postulates that turbidity currents have predominantly governed sedimentation in this region during the Pleistocene. This work describes the first example of contourite depositional system identified in the Vema Fracture Zone. The discovery was made through detailed high-resolution sub-bottom profiling, as well as numerical modeling and direct measurements of bottom current velocities. Such systems are exceptionally uncommon in fracture zones. This study highlights the importance of further research of contourites along the Vema Fracture Zone based on modern concepts of contourite and mixed depositional systems. The work also emphasizes the need to reevaluate the impact of bottom currents on sedimentation in this region, and particularly in the narrow segments of the fracture zone valley.

## Introduction

Contourite deposits in the abyssal realms of the Atlantic serves as the records of the activity of the North Atlantic Deep Water (NADW) and Antarctic Bottom Water (AABW)^1,2^ which are important parts of the Atlantic meridional overturning. The AABW is distributed in the Atlantic through a series of deep-water passages (e.g., Vema Channel, Kane Gap, Discovery Gap, Romanche Fracture Zone). The contourite depositional systems (CDS) related to these oceanic passageways are key to study the history of the AABW activity and its impact on sedimentation^1,3–7^ (on a scale of hundreds of thousands and millions of years). A family of long-offset fracture zones (FZ) is a distinctive feature of the Central Atlantic morphology^8,9^. They were inherited from the initial break-up of the Atlantic and serve as crucial pathways for the AABW propagation from the western to the eastern part of the ocean^10^. However, this region is still pretty much a blank spot in the modern CDS distribution databases^11–13^. A rare example of a specific study focusing on contourites within the fracture zones of the Central Atlantic is the work by Westall et al.^14^ describing the sediment waves on the flank of the northern transverse ridge that borders the Romanche FZ.

The Vema FZ is one of the most prominent fracture zones in the region. It plays a crucial role in the AABW propagation from the Western to Northeastern Atlantic. The AABW transport surpasses the transport observed in any other fracture zone of the Central Atlantic^15–20^. Despite the intense AABW net flow through the FZ and high sediment input^21–24^, few contourite features were documented in the area. Heezen et al.^21^ revealed ripples, scouring and winnow marks on bottom photographs from the fracture zone valley. Kastens et al.^25^ reported on the sinusoidal sedimentary bodies distributed within and close to the Principal Transform Deformation Zone in the eastern part of the active transform valley. The sediment cover in this zone is subject to significant post-depositional deformations^25^. Nevertheless, the authors preferred the depositional (non-tectonic) hypothesis for the formation of these features and interpreted them as buried sediment waves.

Active studies of the sediment cover within the Vema FZ took place during the dominance of the turbidite paradigm^22,25–28^, which became the primary paradigm for deep-sea sedimentation in the region. It is widely accepted that the sedimentation within the Vema FZ valley was mainly controlled by gravity flows from the South American continental slope (at least during the Pleistocene)^21,27,29^. This conclusion is largely based on studies that focused on the active part of the fracture zone between the two ridge-transform intersections (RTIs)^21,22,25–27,30^. This work explores an understudied sector of the Vema FZ located east of the eastern RTI. It reports on the discovery of a depositional system comprising moats and drifts within the fracture zone valley. This represents a remarkable and infrequent occurrence in the Atlantic Ocean (a few more examples are described by Uenzelmann-Neben and Gohl^31^, Scrutton and Stow^32^), which is important for further development of contourite drift classification and for broadening the understanding of the environments in which drifts can form. The study is based on the results from the analysis of high-resolution sub-bottom profiles and from numerical modeling of bottom current velocities carried out in the investigated part of the fracture zone for the first time. The results presented herein allow a new perspective on sedimentation in the study area, viewed through the lens of modern conceptual paradigms for contourite and mixed depositional systems (clearly summarized by Hernández-Molina et al.^33^). However, this work does not deny the significant contribution of turbidity flows from the South American slope to the sedimentary infilling of the fracture zone valley.

## Regional settings

### Sea-floor morphology

**Figure 1:**
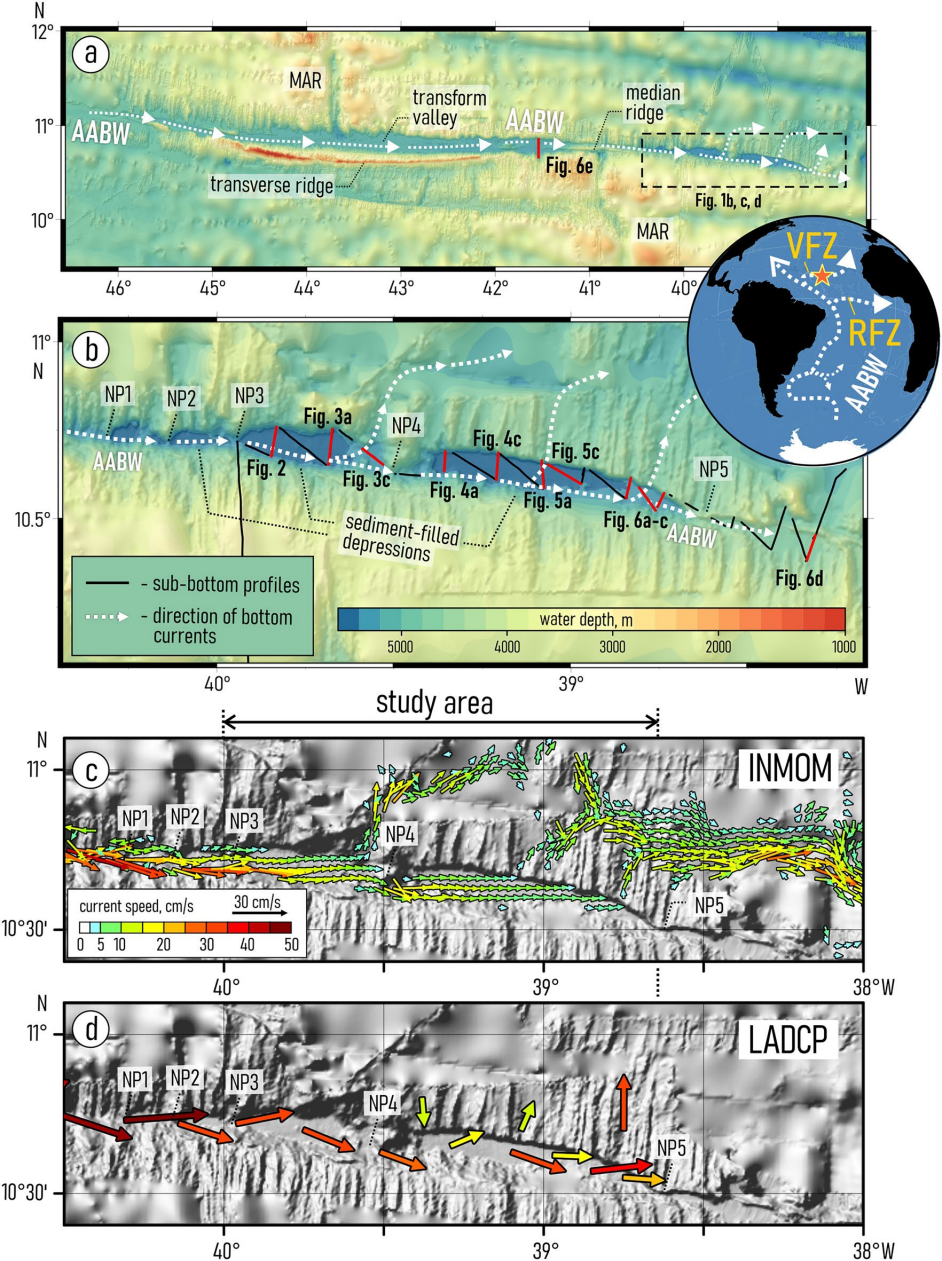
(**a**) The general bathymetry of the Vema Fracture Zone^36^ with the general directions of the AABW current^19^; dashed square marks the location of the study area, red lines mark sub-bottom profiles presented in this work. (**b**) Bathymetric scheme of the study area^36^ with location of the sub-bottom profiles and directions of the AABW flow, red lines mark the sub-bottom profiles presented in this work; (**c**) Results of numerical modeling of bottom current velocities in the study area using the Institute of Numerical Mathematics Ocean Model (INMOM); (**d**) Results of direct measurements of bottom current velocities using a Lowered Acoustic Doppler Current Profiler (LADCP). The inset at the top right shows the general directions of the AABW propagation from the SW to the NE Atlantic^19,36^, star marks the location of the study area. *MAR*—Mid-Atlantic Ridge, *VFZ*—Vema Fracture Zone, *RFZ*—Romanche Fracture Zone; *NP1-5*—narrow passages described in the text. Bathymetric schemes from (**a**–**d**) are based on the GEBCO_2022 data set (https://www.gebco.net/data_and_products/gridded_bathymetry_data/gebco_2022/).

The Vema FZ is traced between 52° W and 23° 30′ W and extends for approximately 2300 km along 10° 50′ N (Fig. 1a). It has a 15–20 km wide flat valley (Fig. 1a) at a water depth over 5100 m^21,25^ bounded by a steep slope in the north and by the northern flank of the prominent Vema transverse ridge in the south^10,34^. The thickness of sediments covering the rough basement topography reaches 1.5 km^21,25,26^. It is assumed that the sediments infilling the trough are predominantly turbidites deposited mainly during the Pleistocene^21,27,29^. The turbidity currents entered the Vema FZ from the Demerara Abyssal Plain and brought the continent-derived material into the transform valley. In the east (at ~ 41° W), the transform valley is blocked by a 1.2 km high median ridge which prevents further eastward propagation of gravity flows^22,25,30,35^ (Fig. 1a). This ridge is a notable topographic feature formed within the Vema transform valley near the eastern ridge-transform intersection mainly due to a tectonic uplift of crustal blocks^30^. The age of the median ridge formation remains unclear. It is known that the ridge is not a very recent constructional volcanic feature^30^ and that in the Pleistocene its western end has undergone an uplift at a rate of approximately 1 mm/year^25^.

**Figure 2:**
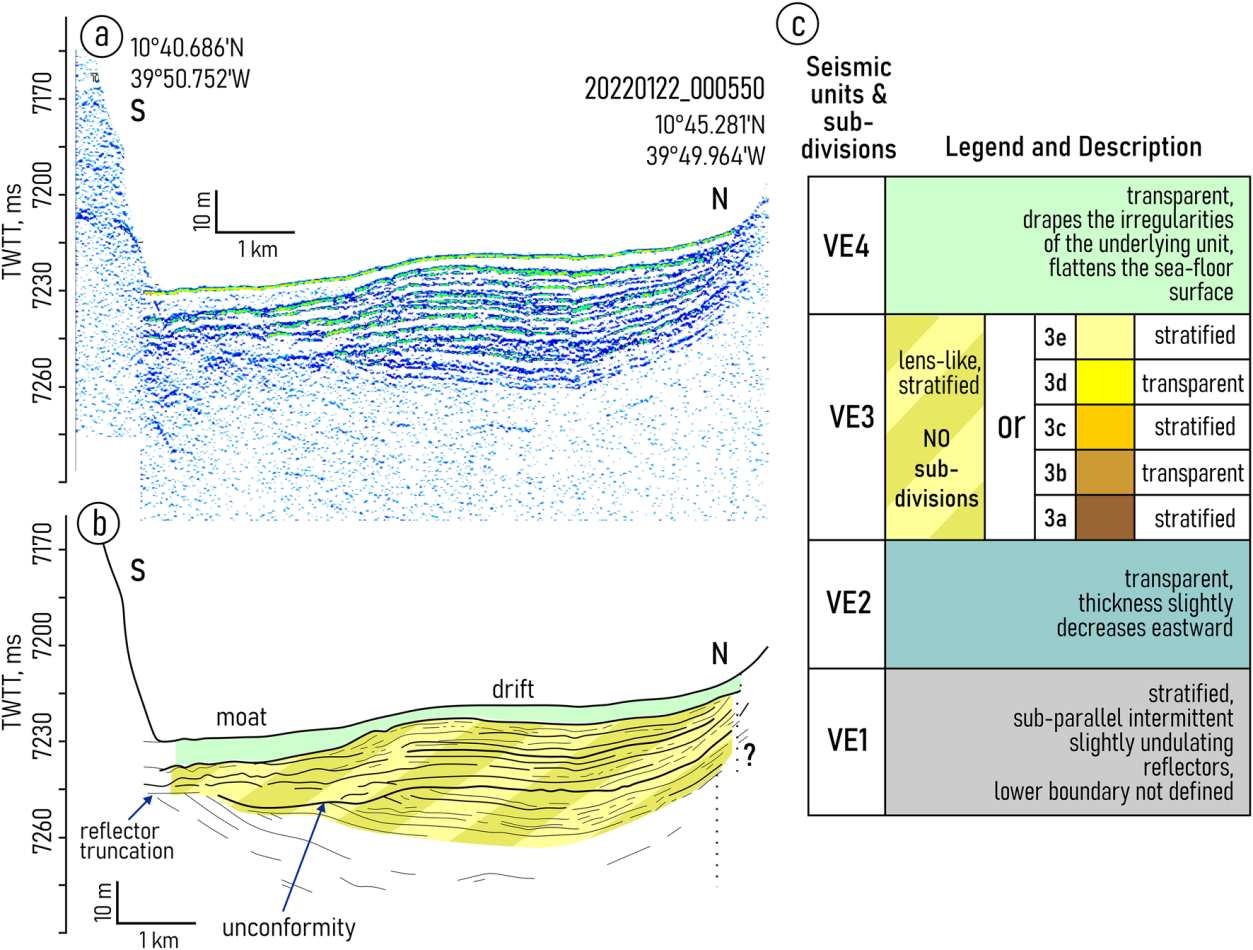
Sub-bottom profiling record across the drift and moat in the western depression (**a**) with interpretation (**b**), location of the profiles is shown in Fig. 1b; (**c**) a summarized description of the acoustic structure of the upper sediment cover in the study area.

The study area embraces a part of the FZ between 40° W and 38° 38′ W (Fig. 1b). The narrowing of the trough in this area formed a series of ellipsoidal depressions filled with sediments and surrounded by steep slopes (walls). The slope angles reach on the southern and northern walls 23° and 45°, respectively^36^. The depressions are connected to each other with small passages located near the southern wall. These passages are numbered from west to east as NP1–NP5. The study area is confined between NP3 and NP5 (Fig. 2b).

The water depth in the passages decreases eastward from 5400 m (NP1, 40° 19′ W) to 4600 m (NP5, 38° 39′ W). The two studied sediment-filled depressions have dimensions of 8.5 × 31.5 km and 8.5 × 78 km. The sediment infill provides a smooth sea-floor surface at a water depth range of 5300–5500 m^36^. The total thickness of sediment cover in these depressions remains unknown. Hereinafter these depressions are referred to as the western (smaller) and eastern (larger) depressions, respectively.

### Oceanographic setting

The bottom circulation in the deepest part of the Vema FZ is controlled by the Antarctic Bottom Water (Fig. 1) underlying the Lower North Atlantic Deep Water (LNADW)^17–19,37–39^. The lack of generally accepted criteria for determining the AABW/LNADW boundary in this area makes the distinction of the depth range occupied by the AABW ambiguous^19,38^. The LNADW is characterized by a local maximum of oxygen and chlorofluorocarbon contents^17^. According to the variations of the dissolved oxygen content in the water column the AABW / LNADW boundary is located in the study area at a depth of approximately 4100 m and corresponds to the isotherm of (potential temperature) θ = 1.7 °C^17,38^. The AABW moves along the Vema FZ in the general eastward direction^19^ (Fig. 1a,b). The direct measurements of current velocity (~ 41° W) closest to the study area revealed strong short-term variations in the direction and speed of the AABW flow (on scale of hours and days)^35,38,39^. The measured current speed reached 20–33 cm/s^19,35,38^. The time averaged velocities can be estimated based on the data collected using moorings with current meters in the Vema FZ^35^. These current meters showed that the flow direction varied strongly and the mean velocities were approximately 2.9 and 3.7 cm/s at the depth of 5040 m, while the maximum observed velocities reached 33 cm/s. Repeated measurements using a Lowered Acoustic Doppler Current Profiler (LADCP)^39^ suggest that the mean velocities are much higher in the narrow sills of the fracture zone. However, only long-term velocity time series can be used for accurate mean velocity calculations. Estimates of the AABW transport through the Vema FZ vary significantly (from 0.05 to 2.4 Sv, 1 Sv on average, according to Vangriesheim^35^, McCartney et al.^15^, Fischer et al.^16^, Demidov et al.^18,38^, Morozov et al.^20^ and others) depending on the adopted depth of the AABW/LNADW boundary, the methods for estimating the square of the section and the extrapolation of the current velocities^19^. The total AABW transport through the group of fracture zones south of the Vema FZ was estimated at 0.48 ± 0.05 Sv^15–20^. The AABW transport to the eastern part of the Atlantic through the fractures north of the Vema FZ is most likely insignificant^15–20^.

## Results

### LADCP and bottom current velocity modeling

Both modeling and direct measurements show the relative acceleration of the AABW flow within the narrow passages and the further slow-down in wider parts of the trough (Fig. 1c,d). The LADCP data show that the flow speed changes from 49 cm/s at the narrow passage labeled NP1 to 31 cm/s at NP2 and 32 cm/s at NP3. In the western depression (between narrow passages NP3 and NP4), the measured bottom current speed reaches 30 cm/s. Current speed decreases down to 26 cm/s at NP4 and to 18 cm/s in the eastern depression.

The numerical model generally confirms these data. In the western depression, the modeled speeds vary from 29 cm/s near the southern wall to 8 cm/s near the northern one. In the eastern depression (between (NP4 and NP5) the modeled bottom flow speed reaches 15 cm near the southern wall slows down to 7 cm/s near the northern wall.

Several outflows of bottom waters through the northern wall of the Vema FZ are observed in the studied part of the fracture zone (Fig. 1c,d). Numerical modeling suggests that significant outflows are located at 39° 33′ W, 39.0° W and 38.7° W. Some part of bottom waters can propagate further east through NP5.

### Sub-bottom profiling

**Figure 3:**
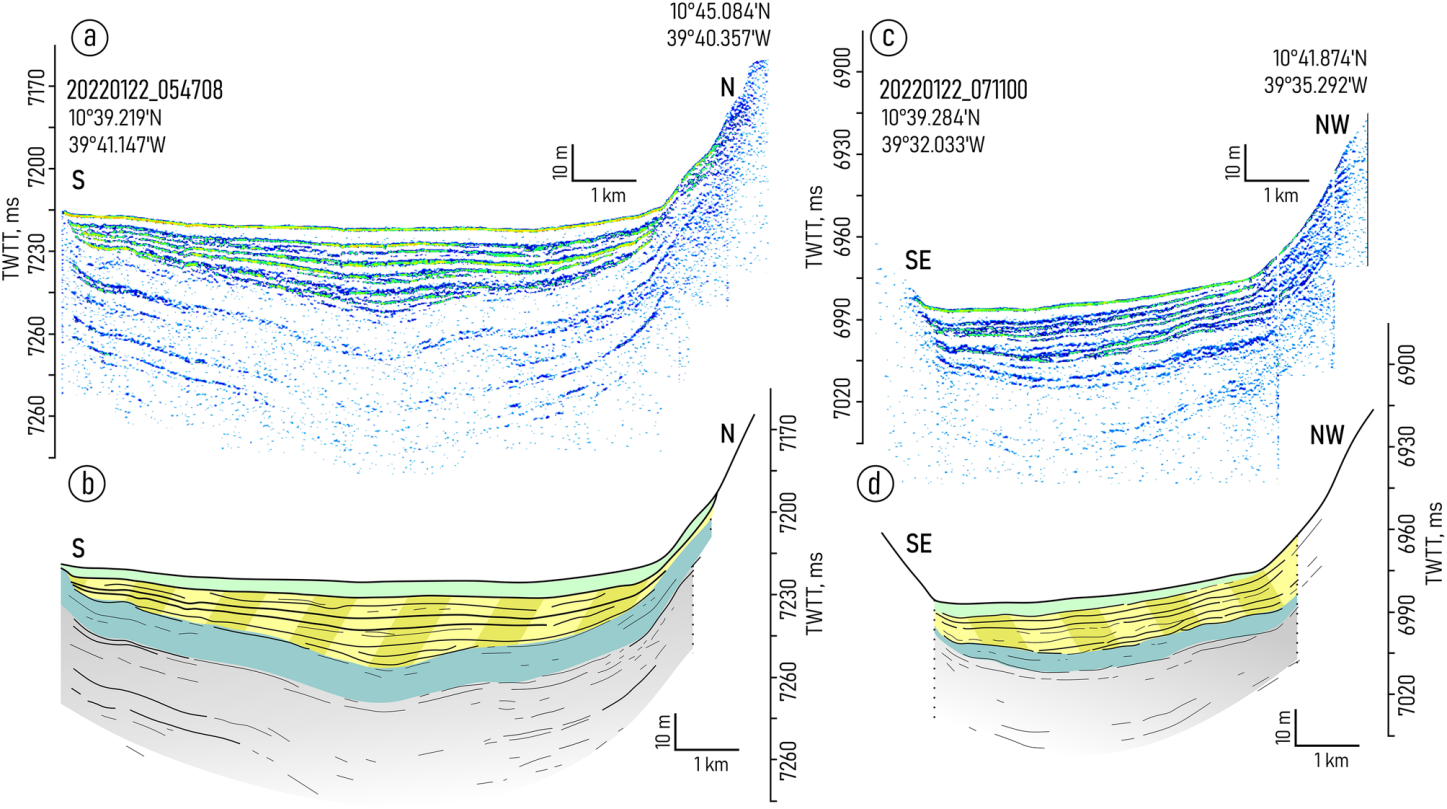
Sub-bottom profiling records from the western depression (**a**,**c**) with interpretations (**b**,**d**), location of the profiles is shown in Fig. 1b. Legend for the interpretation is given in Fig. 2c.

**Figure 4:**
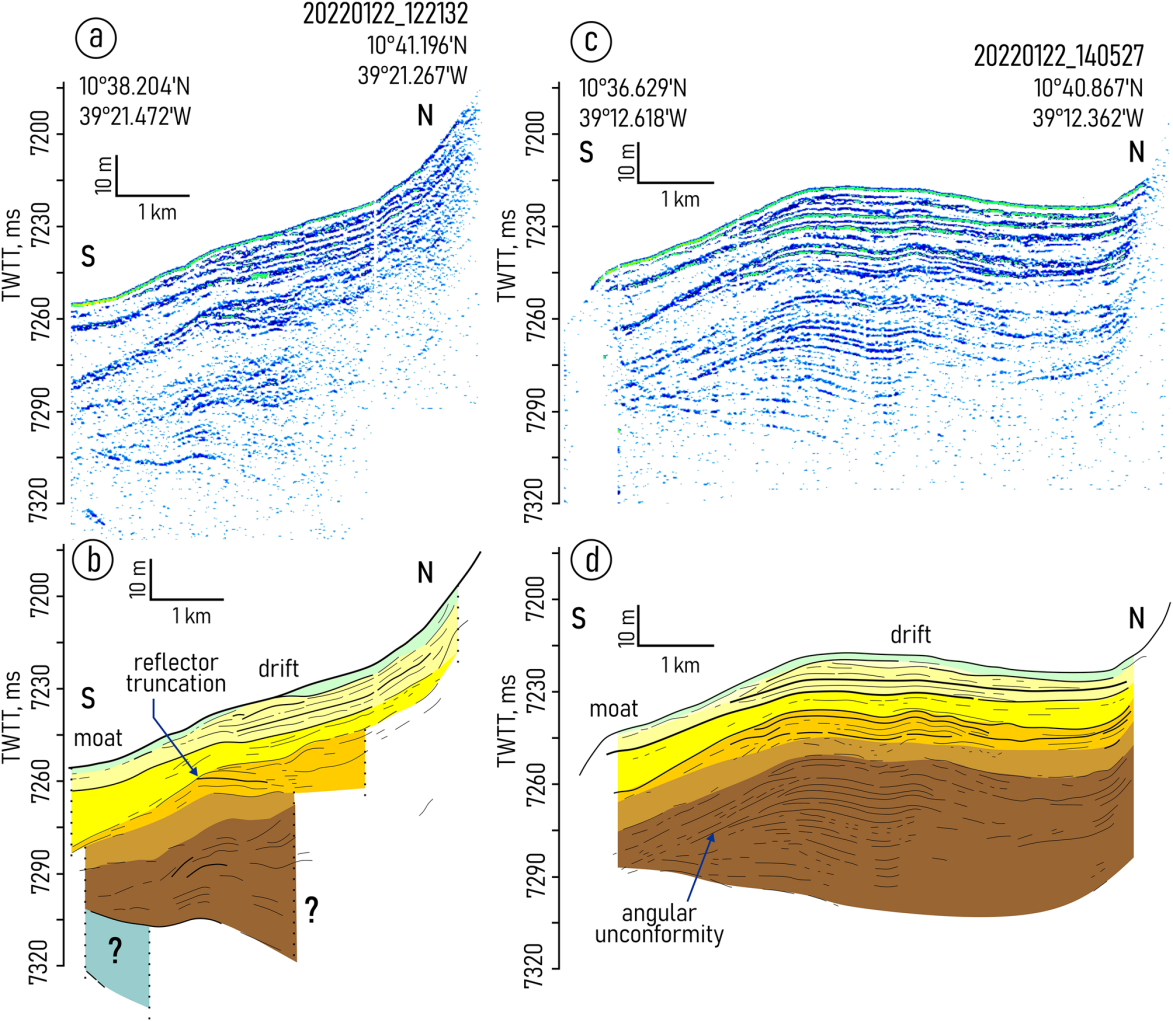
Sub-bottom profiling records across the drift and moat in the eastern depression (**a**,**c**) with interpretations (**b**,**d**), location of the profiles is shown in Fig. 1b. Legend for the interpretation is given in Fig. 2c.

**Figure 5:**
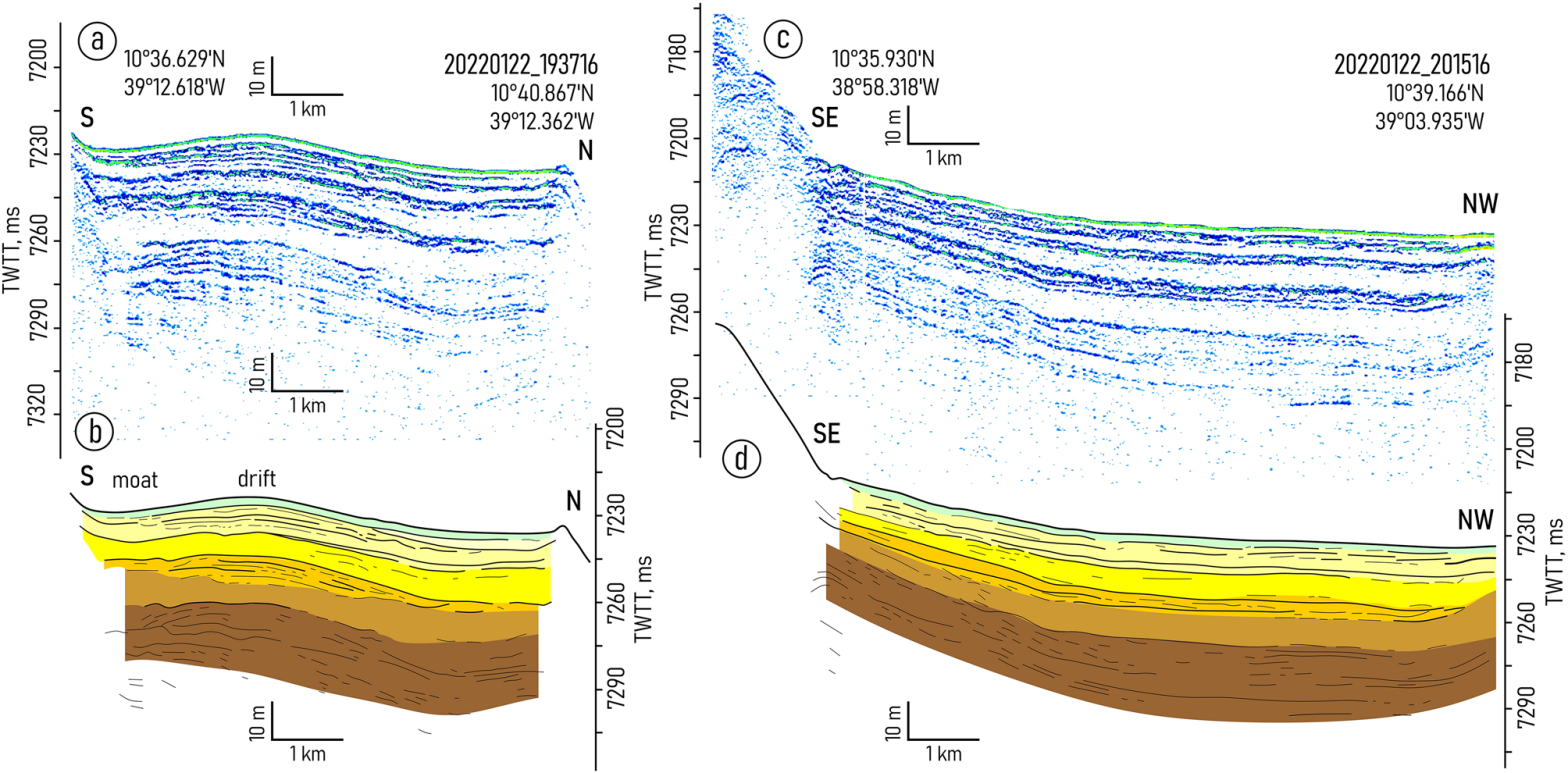
Sub-bottom profiling records from the area of the AABW outflow through the northern wall, eastern depression (**a**,**c**) with interpretations of these records (**b**,**d**), location of the profiles is shown in Fig. 1b. Legend for the interpretation is given in Fig. 2c.

**Figure 6:**
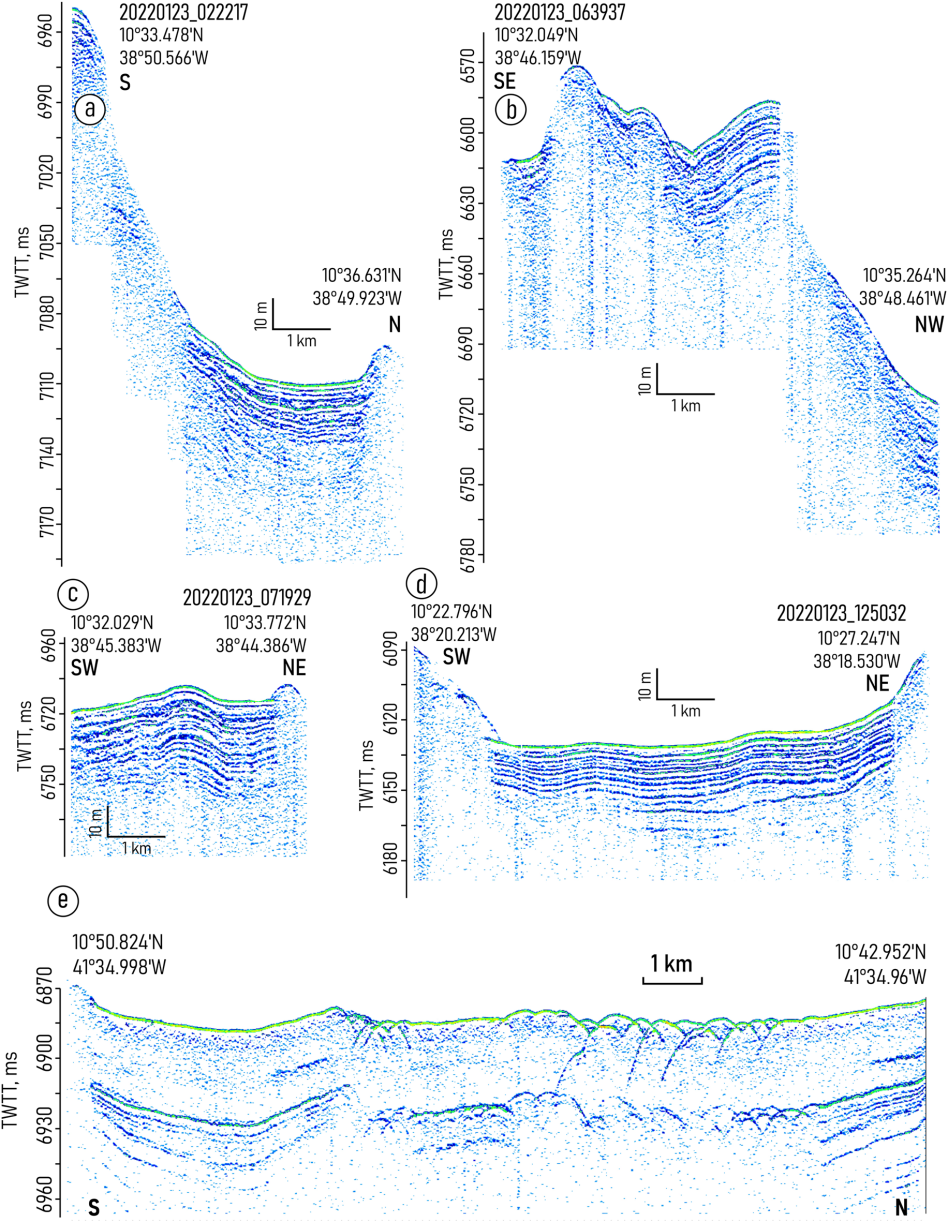
(**a**–**d**) Sub-bottom profiling records in the areas outside of the contourite depositional system boundaries; (**e**) sub-bottom profiling record showing the effect of tectonic activity on the sediment cover structure in the Vema transform valley. Location of the profiles is shown in Fig. 1a,b.

Both depressions embraced in the study are characterized by the similar changes in the sediment filling pattern. From west to east, the mounded onlap fill pattern gradually transforms into a divergent fill (Figs. 2, 3, 4, 5 and 6, Supplementary Figs. S2–4). Two linear channel-like topographic features with a depth of approximately 5–20 m and a width of 1–3 km extend along the foot of the southern wall (Figs. 2a,b, 4). It can be traced between 39° 54′ and 39° 42′ W in the western depression and between 39° 21′ W and 39° 03′ W in the eastern one. The relative depth of the features decreases gradually eastward from narrow passages NP3 and NP4, respectively (Figs. 4, 5).

The structure of the upper sediment cover in the study area is subdivided into four seismic units (VE1–4) numbered from bottom to top and described here in the same order (Fig. 2). The lowermost unit VE1 was traced only in the western depression (Fig. 3). The unit shows the acoustic stratification with sub-parallel intermittent slightly undulating reflectors. The lower boundary of the unit was not defined due to insufficient acoustic penetration. The upper boundary is mainly indistinct. In the western depression, close to NP3, there is evidence of a reflector truncation at the top of the unit (Fig. 2a,b). The truncation is observed only near the southern wall.

The unit VE2 is uniform and almost acoustically transparent with a thickness of 8–14 ms two-way travel time (TWTT). From its top it is bounded by an angular unconformity. The unit can be recognized mainly in the records from the western depression (Fig. 3).

The overall geometry of the unit VE3 changes similarly in both depressions studied. The lens-like upward-convex geometry in the west changes to downward-convex geometry in the east (Figs. 2a,b, 3, 4, 5). The unit thickness shows lateral variations. It decreases from the axis of the trough toward the walls. In the eastern depression the unit is twice as thick as in the western one (80 ms and 40 ms, respectively) (Figs. 2a,b, 4). The internal reflection patterns of the unit VE3 show variations both in vertical and lateral directions (N-S and W-E). The vertical structure of the unit is characterized by intercalation of stratified and relatively transparent sub-units bounded by unconformities (Figs. 4, 5). Five sub-units are numbered from bottom to top as VE3a-e (Fig. 2c). The sub-units were revealed in the western part of the eastern depression and become indistinguishable in the area where the thickness of unit VE3 decreases. Transparent sub-units increase in thickness and prevail in the vertical structure mainly near the southern wall (Fig. 4). However in the areas where bottom water turns to the north and leaves the trough at 39.0° W (Fig. 1c,d), the acoustically transparent deposits increase in thickness at the foot of the northern wall (Fig. 5). Stratified sub-units demonstrate a progradational pattern with internal angular unconformities and reflector truncations (Fig. 4) while the entire unit VE3 is generally aggradational.

The uppermost unit VE4 shows no internal reflections. It drapes the irregularities of the underlying unit and flattens the sea-floor surface. The unit thickness decreases eastward from 6 ms in the smaller western depression to less than 1.5 ms (TWTT) in the eastern one.

Eastward of NP5, the sub-bottom profiling recorded mainly acoustically stratified deposits without evidence of the units described above (Fig. 6d).

## Discussion

Tectonic processes significantly affected the structure of the sediment cover to the west of the median ridge, in the active Vema transform valley^25,26,40^ (Fig. 6e, Supplementary Fig. S5). The post-depositional deformations can be recognized there even on the ocean-floor surface^25,26^. However in the upper part of sediment cover in the studied depression, the analysis of sub-bottom profiling data did not reveal any evidence of faulting or deformations caused by tectonic activity.

**Figure 7:**
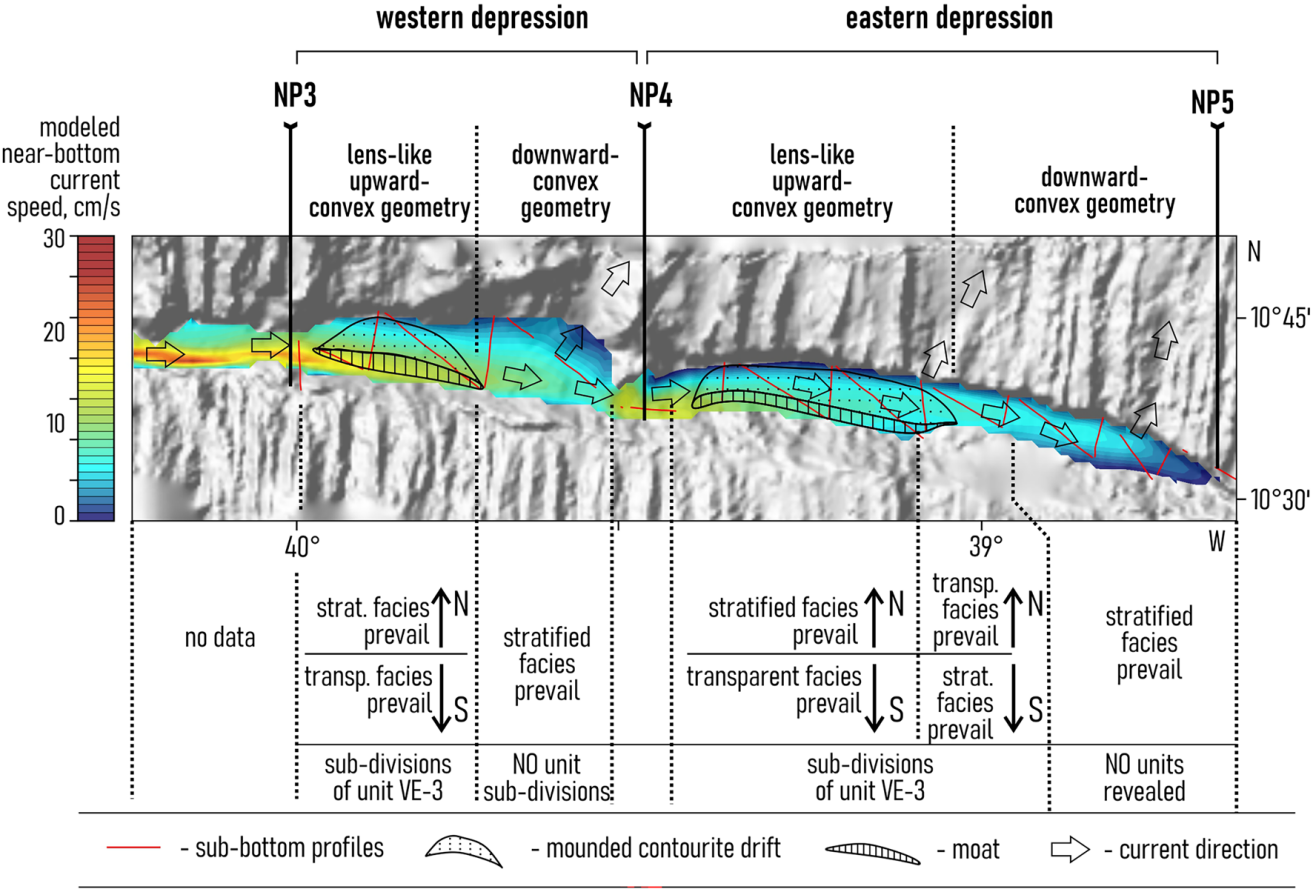
The location of the drifts and moats superimposed on the distribution map of the modeled bottom current speeds, with summarized characteristics of seismic structure of the upper sediment cover. Bathymetric scheme of the study area is based on the GEBCO_2022 data set^36^ (https://www.gebco.net/data_and_products/gridded_bathymetry_data/gebco_2022/).

The median ridge prevented the eastward propagation of the gravity flows moving from the South American continental slope to the transform valley^22,25,30,35^. The age of the median ridge formation remains uknown^25,26,30^. What is known however, is that the ridge existed in the Pleistocene^25,30^. Due to the isolation from the direct impact of the turbidity currents from the continental slope, the sedimentation in the study area was controlled by the interplay between current-related processes, pelagic settling and gravity flows from the northern and southern walls. Heezen et al.^21^ suggested that in the region to the east of the median ridge, gravity flows could transport calcareous material from the walls to the valley. Despite the ocean depth of over 5000 m and the generally high capacity of AABW to dissolve calcium carbonate, the calcareous material in the Vema FZ forms layers of tens to hundreds of centimeters in thickness^21^. The sea-floor morphology in the study area significantly affects the velocity of the bottom currents which in turn affect the sedimentation. The median ridge as a prominent topographic obstacle causes an increase in the bottom current velocity due to the narrowing and shallowing of the valley. The accelerated bottom water flow maintains high speeds because of the low rates of kinetic energy dissipation and relative acceleration at the narrow passages (Fig. 7). The same effect has been observed in other fracture zones of the Atlantic^39,41^.

**Figure 8:**
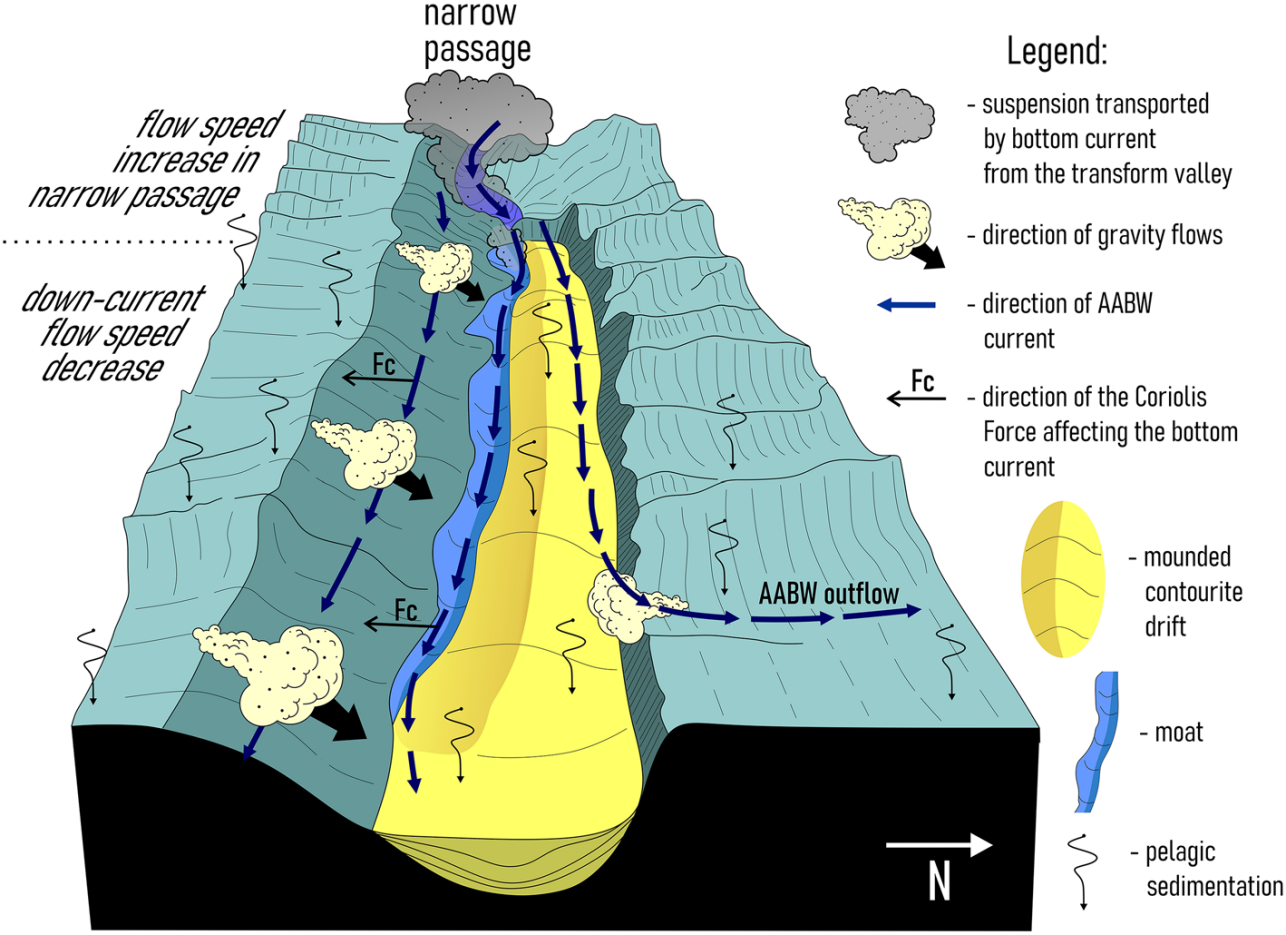
A sketch showing the interplay between the main sedimentation processes in the study area.

The AABW current could help in the distribution of the terrigenous material provided by gravity flows along the transform valley and transport the fine suspended sediment material over the median ridge to the study area (Fig. 8). The fluctuations in the bottom current velocity and the supply of terrigenous material could result in the formation of the deposits with contrasting variations in composition, density, acoustic impedance. Consequently, these deposits exhibit a stratified structure in sub-bottom profiling records. The acoustically transparent sediments are suggested to have a more homogeneous mostly calcareous composition (due to a limited supply of terrigenous material to the study area). Both gravity flows from the walls and pelagic settling brought mainly biogenic calcareous material which (as proposed by Heezen et al.^21^) could be reworked by bottom currents. The cyclic intercalation of acoustically stratified and transparent units most probably occurred in response to the climatic and oceanographic changes (e.g. eustatic sea level changes) rather than to tectonic processes. The fall of the sea level in glacial periods caused intense erosion of the South American shelves^42^ channeling of the Amazon discharge directly to the deep water realms and intensification of gravity-driven processes transporting sediment material toward the Vema FZ^43–45^. Previous studies reported on the enhanced AABW current activity in the Vema Channel and Discovery Gap during glacials and especially at their terminations^1,7^. These passages are located on the way of the AABW before and after the Vema FZ and also play a crucial role in the AABW propagation in the Atlantic. During the interglacials, the sea level and biological productivity increased while the input of the Amazon sediment material to the Deep Ocean and AABW erosion-depositional activity decreased. Nevertheless, all these assumptions about the glacial-interglacial origins of acoustically stratified and transparent deposits do not allow to perform an unambiguous estimation of the unit age.

Units VE1 and VE2 do not show any clear evidence of contourites and served as a substrate for the development of the contourite depositional system that fits in unit VE-3. Within this unit, the channel-like features slightly migrating toward the southern wall have been recognized (one feature in each depression) (Figs. 2a,b, 4b,d). A lateral tracing of these features revealed two small “channels" extended along the foot of the southern wall (Fig. 7). Their location corresponds to the area of the increased values of the modeled bottom current speeds (Fig. 7). The high current velocities near the southern wall are associated with the displacement of the AABW flow to the right of the current direction, which is clearly shown by numerical modeling (Figs. 1c, 7). This important aspect of the abyssal water dynamics occurs in the study area despite of the low latitude and the correspondingly low Coriolis parameter. The importance of the Coriolis force for the structure of bottom waters in the Vema FZ was also shown by Frey et al.^46^. The revealed channel-like features have greater relative depths and widths close to the western parts of the depressions where the accelerated water flow leaves the narrow passages NP3 and NP4, respectively. Further east, the current speed decreases, these features get less expressed in the ocean-floor topography and disappear in the middle part of the depressions. According to the above, these features were interpreted as moats formed under the influence of the AABW current. As their definition suggests, moats should be associated with contourite drifts^11^. The upward-convex lens-like depositional bodies recognized within unit VE3 and adjacent to the moats are considered confined contourite drifts (Figs. 2, 4, 5, 7). This interpretation is supported by the location of the depositional bodies, presence of moats, the mounded geometry (with asymmetric mound and moat), an overall down-current elongation, erosional discontinuities at the base and within the drifts, broadly lenticular, upwardly-convex seismic units, low- to moderate-amplitude, subparallel continuous and intermittent reflectors^47–51^. The transparent and stratified sub-units within the drift structure correspond to different stages of the drift formation. The most intense AABW current activity during the glacial-interglacial transitions could be responsible for the formation of the prominent internal unconformities between the stratified and transparent units and sub-units VE3a/VE3b, VE3c/VE3d, VE3e/VE4 (Figs. 2a, 4b,d). The higher bottom current speed in the western depression, as inferred from the numerical modeling and direct measurements (Fig. 1c,d), caused more active erosion compared to the eastern depression (Fig. 2a,b). This explains the twofold difference in thickness of the studied drifts and less clear evidence of intercalation between stratified and transparent sub-units in the western depression. The erosion by bottom currents could also destroy the evidence of the drift migration due to the widening of the moats (Figs. 2a,b, 4).

The drifts have lateral dimensions of approximately 20 × 7 km and 36 × 6 km in the western and eastern depressions, respectively. The eastern boundaries of the drifts and moats correspond to the decrease in the modeled current speed below ~ 10 cm/s and to the regions where a part of the AABW outflows through the northern wall. It should be noted that the simulated velocity field represents the modern mean climatic values of the current speeds. As mentioned above, in the geological past, the bottom current velocities could be more intense comparing to modern ones. Given that we did not take into account the processes that can be triggered by velocity variations on the synoptic and seasonal scales, the modeled velocities can be quite different from the simultaneous velocities obtained using LADCPs. This feature of the bottom current simulations was discussed in detail in Frey et al.^52^. Anyway, according to the bedform-velocity matrix^53^, 10 cm/s is the lowest current speed limit for the formation of contourite depositional features in an abyssal environment. The bifurcation of the AABW current and the moving of its part through the northern wall could eventually change the flow pattern in the depressions in a way that would hinder the formation of contourite features. The relative increase in the thickness of the transparent units at the foot of the northern wall in the area of the AABW outflow at 39.0° W (Fig. 5) was possibly caused by the activity of gravity-driven processes induced by the bottom currents. The intense pulsating water flow moving through the passages in the steep northern wall could increase the instability of unconsolidated calcareous sediments and trigger turbidity currents (Fig. 8). However in the other sub-bottom profiles near the AABW outflows at 39° 33′ W and 38.7° W there is no clear evidence of activity of gravity-driven sedimentation processes (Figs. 3c, 6b). The relatively fast water flow pushed toward the southern wall by the Coriolis force could work the same way. The sediment material derived from the southern wall and transported by turbidity currents could be reworked by the AABW flow and involved in the drift formation (Fig. 8). It might explain the prevalence of acoustically transparent deposits along the moats at the foot of the southern wall.

The geographic proximity, the similarity of the formation processes and seismic structure allow the discovered drifts and moats to be considered as a contourite depositional system. Vertical settling and gravity flows from the northern and southern walls played a secondary role in the system formation, which is why the system should not be classified as a mixed.

Uniform unit VE4 corresponds to the relatively calm modern stage of the upper sediment cover formation in the study area. This implies that the contourite depositional system is presently inactive. The nature of the deposits in the depressions outside the drifts and moats remains questionable.

The analysis of sub-bottom profiling data combined with the results from the direct measurements and numerical modeling of bottom current velocities revealed that the AABW current played a very important role in the formation of the upper sediment cover sector of the Vema FZ located east of the eastern RTI. The ocean-floor topography significantly affects the structure and velocity of bottom currents in the study area. The diverse impact of the AABW flow on sedimentation includes (1) the formation of the mounded drifts and moats; (2) the triggering of the gravity flows from the steep walls surrounding the studied depressions and subsequent reworking of the material transported by these flows; (3) the possible transport of the suspended fine terrigenous material from the transform valley to the study area. The current-related processes are suggested to be dominant during the glacials and especially during the transitions to the interglacials. LADCP measurements combined with numerical modeling of bottom current velocities revealed the areas of AABW outflows through the northern wall.

The discovered drifts and moats are considered to be the first example of contourite depositional system in the Vema Fracture Zone. This study rediscovers the Vema Fracture Zone as a promising target for contourite research and provides a basis for reconsidering the contribution of bottom currents to sedimentation in other parts of the fracture zone where the valley narrows.

## Data and methods

### LADCP measurements and bottom current modeling

Two approaches have been combined for studies of the bottom circulation, numerical modeling (Fig. 1c) and direct in situ measurements (Fig. 1d). The numerical simulations provide a three-dimensional velocity field with a high horizontal and vertical resolution, while the direct measurements using a Lowered Acoustic Doppler Current Profiler (LADCP) allow the validation of the model and give accurate current velocity data at several key points along the bottom flow.

The Institute of Numerical Mathematics Ocean Model (INMOM) was used for simulations of currents in the bottom layer of the Vema FZ. The INMOM is a σ-coordinate ocean circulation model based on the primitive equations of ocean hydrothermodynamics with the Boussinesq and hydrostatic approximations^54^. The model was adjusted for the region of the Central Atlantic including the entire Vema FZ (Fig. 1c). The approach used to simulate the bottom currents is described in detail in Frey et al.^55,56^. According to their model, the three-dimensional velocity field was interpolated to a set of 31 constant depth values (from 4000 to 5500 m with a step of 50 m). The analysis of all the results of the numerical modeling, including those near the sea floor, leads to the conclusion that current velocities at a depth level of 4800 m is the best illustration of the currents in the narrow, relatively shallow sills as well as in the wider and deeper parts of the trough (Fig. 1c).

The direct velocity measurements were performed in a cruise of the RV *Akademik Boris Petrov* (2022)^57^ using a Lowered Acoustic Doppler Current Profiler TRDI WorkHorse Monitor 300 kHz mounted on a GO1018 water sampler together with a CTD probe *Idronaut Ocean Seven 320p* (Fig. 1d). The LADCP measurements were carried out from the ocean surface to the depth of 5 m above the sea-floor. The LADCP data processing was performed using the standard LADCP LDEO software version IX.10 described by Visbeck^58^. The results of the processing were adjusted by subtracting the tidal velocities based on the TPXO9 model^59^.

### Sub-bottom profiling

The high-resolution sub-bottom (seismoacoustic) profiles were collected during the RV *Akademik Ioffe* cruise 60 (2022)^60^. The survey was carried out using the *SES 2000 deep* narrow-beam parametric sub-bottom profiler with the center frequency of 4 kHz. Stacking, median filtering and heave correction were applied to the data using the *Interactive Sediment Layer Editor* software. The location of the profiles is shown in Fig. 1a,b and Supplementary Fig. S1. The *SES 2000 deep* profiler provided a high vertical resolution of the acquired records (~ 0.4 m) and acoustic penetration in the study area of down to 80 ms two-way travel time—TWTT (~ 60 m). Additionally, the high resolution seismic line VEMA-07M acquired during cruise VEMA-98 with R/V *Akademik Nikolay Strakhov* across the active Vema transform valley^9^ was used for demonstration of the effect of tectonic processes on the sediment structure within the transform valley (Supplementary Figs. S1, S5).

The sub-bottom profiles and results of numerical modeling in this study are superimposed on the GEBCO_2022 global relief model^36^.

### Supplementary Information

Supplementary Figure S1.

Supplementary Figure S2.

Supplementary Figure S3.

Supplementary Figure S4.

Supplementary Figure S5.

Supplementary Legends.

## Data Availability

All results of the numerical simulations can be accessed through Pangaea (https://doi.pangaea.de/10.1594/PANGAEA.907919). High-resolution sub-bottom profiling records are available through Mendeley (https://data.mendeley.com/datasets/yxd2478rkm/1).
