# Supplementary figures and images for: Unveiling the contourite depositional system in the Vema Fracture Zone (Central Atlantic)
Source: Sci Rep. 2023 Aug 24;13:13834. doi: 10.1038/s41598-023-40401-4 (PMC10449790; doi:10.1038/s41598-023-40401-4)

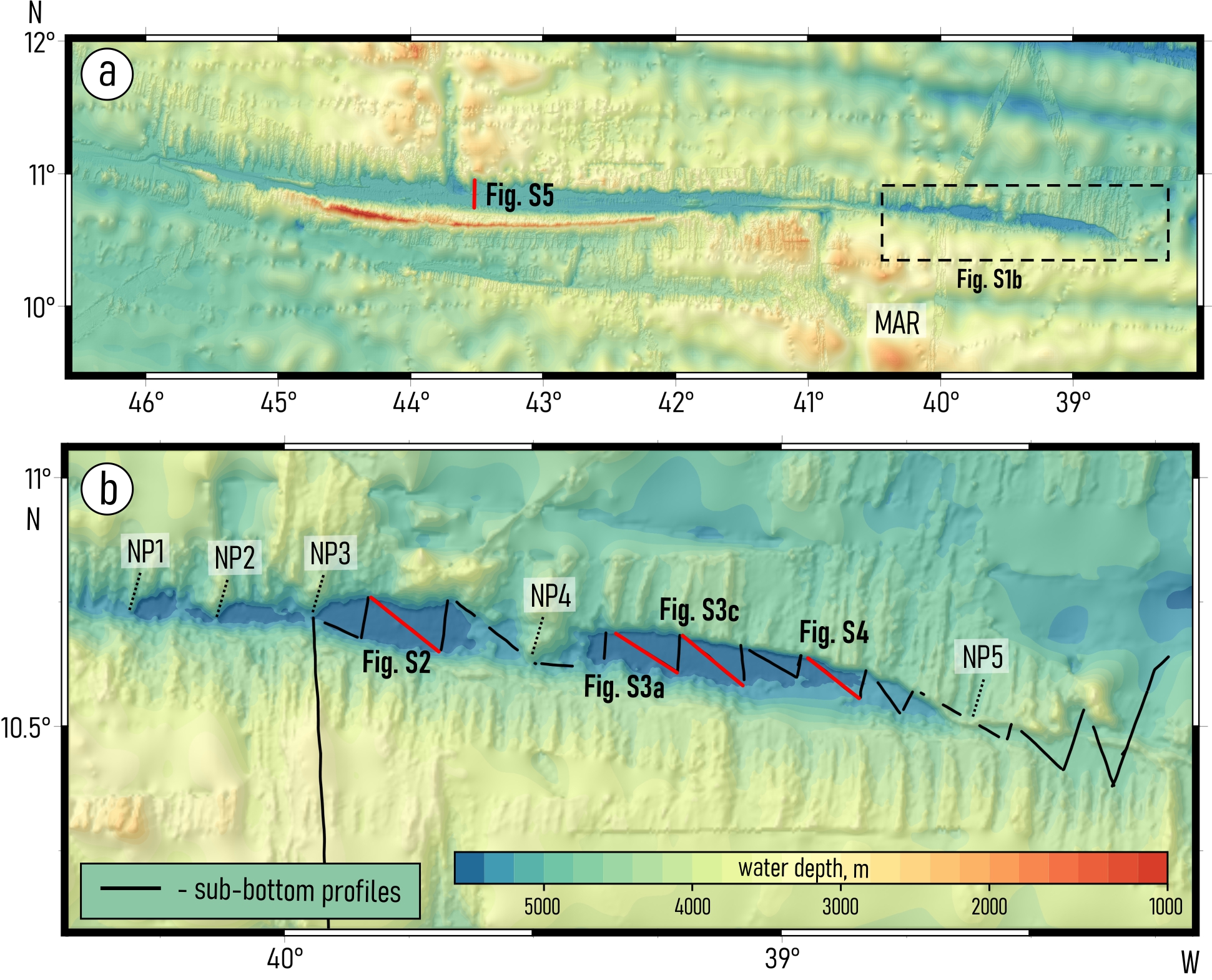

Supplement: Supplementary file 1 — Supplementary Figure S1. [file 41598_2023_40401_MOESM1_ESM.jpg]

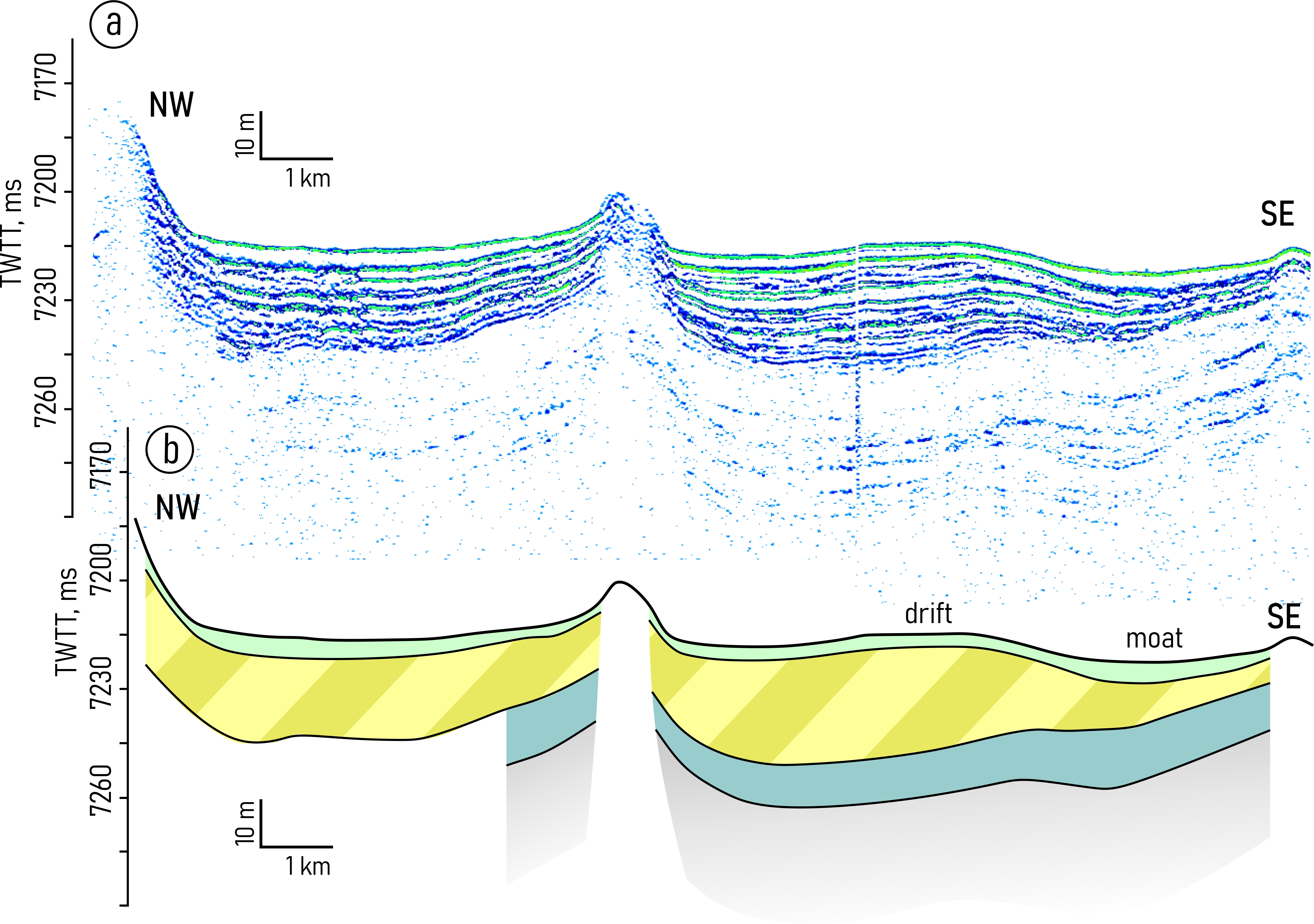

Supplement: Supplementary file 2 — Supplementary Figure S2. [file 41598_2023_40401_MOESM2_ESM.jpg]

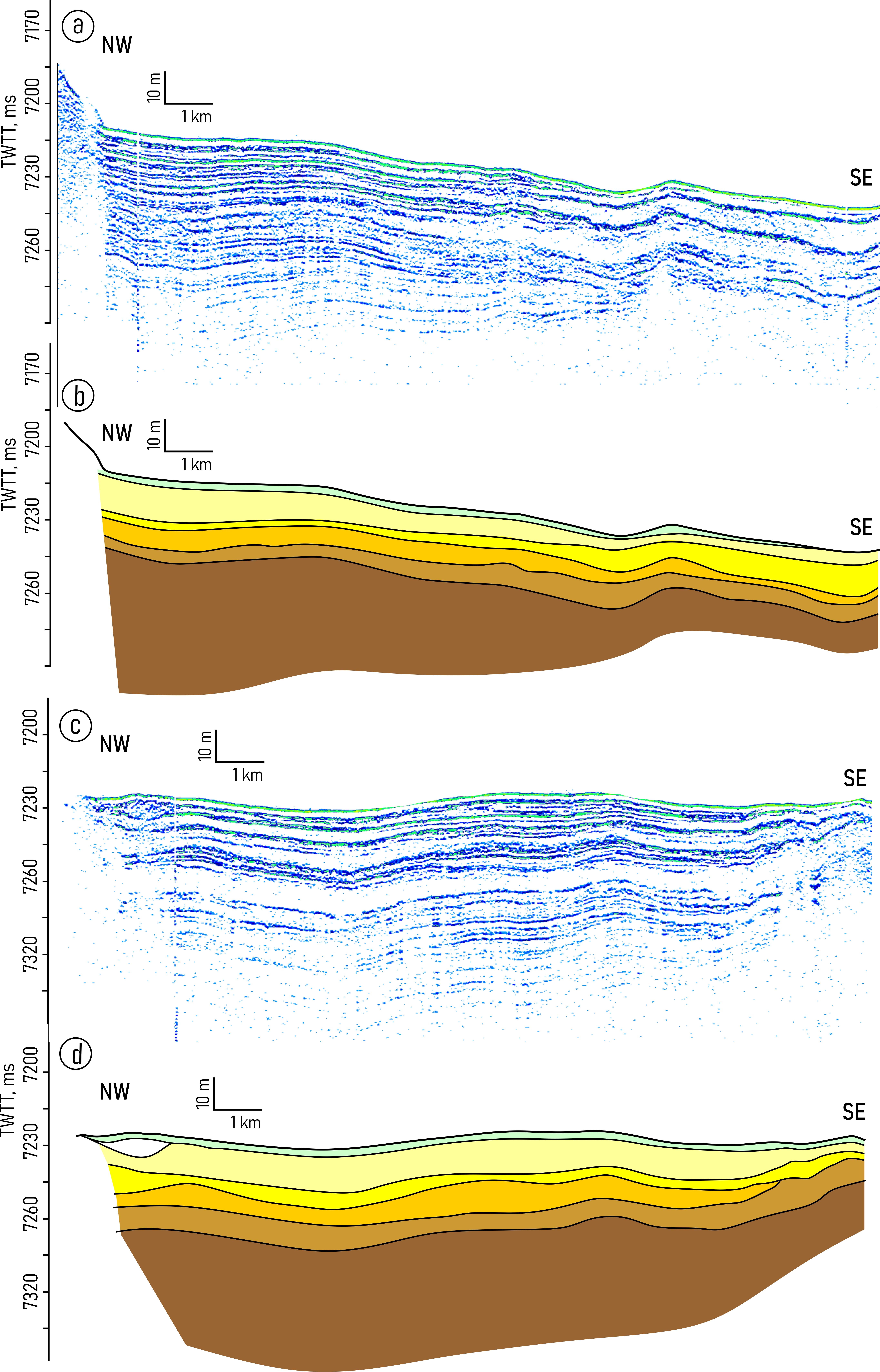

Supplement: Supplementary file 3 — Supplementary Figure S3. [file 41598_2023_40401_MOESM3_ESM.jpg]

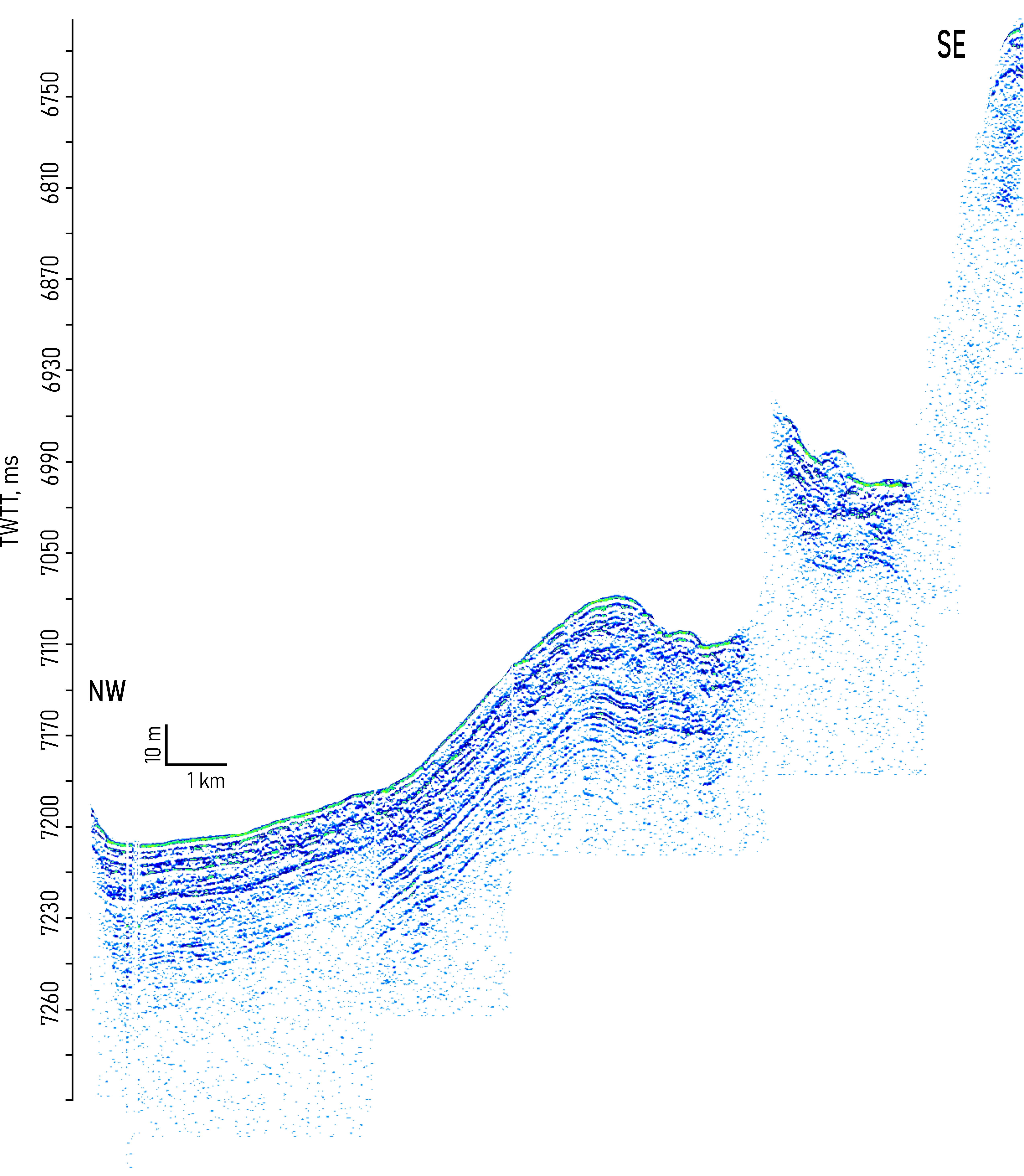

Supplement: Supplementary file 4 — Supplementary Figure S4. [file 41598_2023_40401_MOESM4_ESM.jpg]

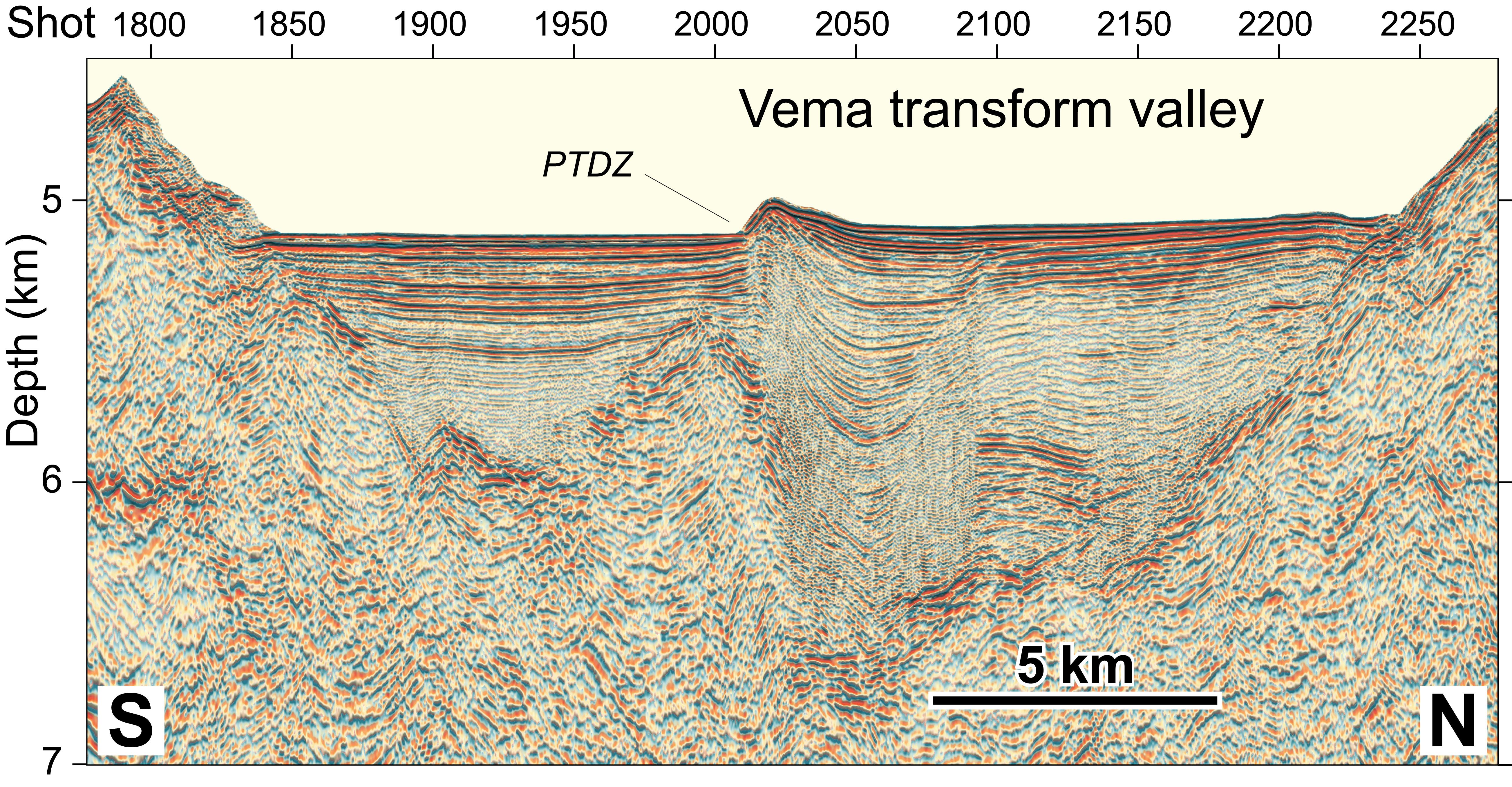

Supplement: Supplementary file 5 — Supplementary Figure S5. [file 41598_2023_40401_MOESM5_ESM.jpg]
