# Supplementary material for: Unveiling the contourite depositional system in the Vema Fracture Zone (Central Atlantic)
Source: Sci Rep. 2023 Aug 24;13:13834. doi: 10.1038/s41598-023-40401-4 (PMC10449790; doi:10.1038/s41598-023-40401-4)
Supplement: Supplementary file 6 — Supplementary Legends. [file 41598_2023_40401_MOESM6_ESM.docx]

**Supplementary Fig. S1.** Bathymetric scheme of the study area^36^ with location of a) the high resolution seismic profile and b) sub-bottom profiles, red lines mark the profiles presented in the Supplementary materials. Bathymetric schemes of the study area are based on the GEBCO_2022 data set (https://www.gebco.net/data_and_products/gridded_bathymetry_data/gebco_2022/).

**Supplementary Fig. S2.** a) Sub-bottom profiling record across the drift and moat the western depression with interpretations (b), location of the profiles is shown in **Supplementary Fig. S1**. Legend for the interpretation is given in **Fig. 2c**.

**Supplementary Fig. S3.** Sub-bottom profiling records across the drift and moat the eastern depression (a, c) with interpretations (b, d), location of the profiles is shown in **Supplementary Fig. S1**. Legend for the interpretation is given in **Fig. 2c**.

**Supplementary Fig. S4.** a) Sub-bottom profiling record in the area outside of the contourite depositional system boundaries, location of the profile is shown in **Supplementary Fig. S1**.

**Supplementary Fig. S5.** Pre-stack ﬁnite-difference depth migration of high resolution seismic line VEMA-07M acquired during cruise VEMA-98 with R/V Akademik Nikolay Strakhov across the active Vema transform valley^44,59^. The transform dynamics strongly influence the sedimentary filling of the valley; PTDZ – Principal Transform Deformation Zone. The location of the profile is shown in **Supplementary Fig. S1**.
